# Canola/Rapeseed Protein: Future Opportunities and Directions—Workshop Proceedings of IRC 2015

**DOI:** 10.3390/plants5020017

**Published:** 2016-04-13

**Authors:** Lisa Campbell, Curtis B. Rempel, Janitha P.D. Wanasundara

**Affiliations:** 1 Canola Council of Canada, 400-167 Lombard Avenue, Winnipeg, MB R3B 0T6, Canada; campbell@canolacouncil.org; 2 Department of Food Science, Faculty of Agricultural and Food Sciences, University of Manitoba, Ellis Building, Winnipeg, MB R3T 2N2, Canada; rempelc@canolacouncil.org; 3 Agriculture and Agri-Food Canada, Saskatoon Research and Development Centre, 107 Science Place, Saskatoon, SK S7N 0X2, Canada

**Keywords:** Canola/rapeseed, protein, fibre, cruciferin, 11S protein, napin, 2S protein, antinutrients, phenolics, phytates, commercial meal

## Abstract

At present, canola meal is primarily streamlined into the animal feed market where it is a competitive animal feed source owing to its high protein value. Beyond animal feed lies a potential game-changer with regards to the value of canola meal, and its opportunity as a high quality food protein source. An economic and sustainable source of protein with high bioavailability and digestibility is essential to human health and well-being. Population pressures, ecological considerations, and production efficiency underscore the importance of highly bioavailable plant proteins, both for the developed and developing world. Despite decades of research, several technologies being developed, and products being brought to large scale production, there are still no commercially available canola protein products. The workshop entitled “Canola/Rapeseed Protein—Future Opportunities and Directions” that was held on 8 July 2015 during the 14th International Rapeseed Congress (IRC 2015) addressed the current situation and issues surrounding canola meal protein from the technological, nutritional, regulatory and genomics/breeding perspective. Discussions with participants and experts in the field helped to identify economic barriers and research gaps that need to be addressed in both the short and long term for the benefit of canola industry.

## 1. Introduction

The Food and Agriculture Organization (FAO) of the United Nations has projected that global food demand, particularly meat and dairy will more than double by 2050. Protein has been identified as a limiting macronutrient for global food security and FAO has stated that sufficient protein quantity and adequate protein quality (bioavailability) are a fundamental right of every global citizen. *“As the world’s population increases rapidly and against the constraints of limiting land, water and food resources, it is more important than ever to be able to define accurately the amount and quality of protein required to meet human nutritional needs.”* While there is an increasing demand for animal-derived proteins (meat, eggs, dairy (casein, whey)) population pressures, ecological considerations and efficiency suggest a rational evolution from animal to plant protein sources for human nutrition. The nutritional value of plant protein is thus of critical importance and is expected to grow in importance globally [1].

Canola/rapeseed (hereafter referred as canola), the second largest produced oilseed in the world after soybean, produces protein-rich meal during oil extraction [2,3]. At present, canola meal is primarily streamlined into the animal feed market where it is a competitive animal feed source owing to its high protein value. Moving beyond monogastric and ruminant animal nutrition and into aquaculture, food, and bioproducts is feasible, but requires research and technology transfer in order to gain commercial acceptance. Canola protein has an opportunity as a high quality human protein source, and an economic and sustainable source of protein with high bioavailability and digestibility. Despite decades of research, several technologies being developed, and products being brought to large scale production, there are still no commercially available canola protein products.

The workshop entitled “Canola/Rapeseed Protein—Future Opportunities and Directions” that was held on 8 July 2015 during the 14th International Rapeseed Congress (IRC 2015), consisted of presentations on four key areas of canola protein and subsequent discussion and elaboration in breakout groups. Presentations addressed the current situation and issues surrounding canola meal protein from the technological, nutritional, regulatory and genomics/breeding perspective with the aim of helping to identify economic barriers and research gaps that need to be addressed in both the short and long term for the benefit of canola industry. This paper presents the information that was presented and discussed during this workshop.

## 2. Summary of Presentation Topics

### 2.1. Processing

Protein product preparation from defatted canola meal has been mostly by aqueous extraction rather than dry fractionation. Aqueous processing of canola protein poses several unique challenges over other plant (seed) proteins. The first of these is the presence of seed phenolics. If phenolic oxidation in the aqueous medium is not avoided, the resulting extracts and final protein products have negative colors and flavors because of phenolic–protein interactions. The second challenge is the protein yield from canola meal. Canola protein has low solubility around neutral pH, therefore alkaline pH or additives such as salt are needed to improve protein recovery yields. The yield of canola protein compared to the total cost of production is known to be less than soy. Canola’s relatively low protein content (36% *versus* 48% in soybean meal) can make protein yields too low to be economical. The third challenge is the processing modifications that canola meal protein receives during oil extraction. Conventionally processed canola meal which has gone through a desolventizer-toaster is an extensively process-modified starting material to recover protein. Expeller pressed meal may be too high in residual oil to enable efficient extraction of protein and would require an extra step of oil removal. Therefore the majority of canola meal available is not the most suitable feed stock material for protein recovery.

Dry processing/fractionation may be applied to defatted oilseed meals, milled pulses and flours, and generally allows the concentration of proteins in the range of 45% to 55%. This is a lower cost approach than wet processing. Canola is known to contain phenolics, glucosinolates, phytates and fibre that are considered as antinutrients. Several of these non-protein compounds are concentrated in the seed coat or hulls. Challenges in dry processing of canola include limited success in removing anti-nutrients, difficulty in hull removal, and integration of these steps in the existing meal preparation and processing schemes. Dry fractionation of canola generates protein-enriched fractions of lower quality flavor, higher microbial load, and lower protein yield than wet processing.

Protein concentrates may also be prepared by acid or ethanol leaching, and this is the typical wet process for soybean protein concentrates. However, in comparison to soybean processing, protein concentrate preparation from defatted canola meal results in lower yield, darker color and lower purity, which is in part due to lower protein content of the meal [4,5].

Preparing protein isolates through alkali extraction and isoelectric precipitation is also commonly used for soybean, and has been tested in canola. Canola protein has lower yield in comparison to soy, possibly due to a wider range of isoelectric points of the constituent proteins. Canola protein isolates may also be prepared through ultrafiltration/diafiltration of aqueous protein extracts. Although co-extracted phenolics, free phytates, and glucosinolates and their breakdown products can be removed to a reasonable extent by this process, challenges for canola may include selection of membranes for effective separation, high water usage and high unit cost. Overall, wet processing of canola protein isolates involves high water usage and energy costs that require prudent economic evaluation for commercial adoption.

Recent advances in technology may improve the yield and quality of canola protein, and also reduce the cost of production. These include centrifuges with reduced energy requirements and higher G-forces for more effective separation which result in lower cost, higher purity, and higher quality protein. Enzyme-assisted, chemical-free processes may allow for extraction in the presence of oil (such as from expeller-pressed meal). The fact that there are no solvents or chemicals used would allow for a clean label, but the process cost may still be a challenge.

A few commercial ventures, including Burcon NutraScience, have overcome a number of these challenges. In Burcon’s process, aqueous extraction, combined with membrane filtration produces canola protein products with distinct functionalities. They are able to produce three different canola protein isolates with excellent functionality and a neutral flavor using a clean and gentle extraction process.

Overall, for any of the above processes the largest challenge facing canola is heat damage to the proteins in the oil extraction process, and especially in the desolventizer/toaster. Heat damage affects the solubility, flavor and color of the protein. Cold pressing and low temperature desolventizing offer a possible solution. If protein products are to become part of the canola value chain, the existing oil extraction process would require some adaptations to accommodate less heat damage to proteins.

### 2.2. Functionality

The functionality of a protein (including the nutritional properties) determines its quality and applicability in food products. The physical and chemical properties of proteins affect their behavior within the environment (usually a food system) during processing, storage and consumption. The functional properties of canola proteins depend on the type of processing, and also on the molecular nature of each of the component proteins. This means the functional properties of different canola protein products may vary depending on the type of proteins recovered as well as the type of processing involved in the production.

Canola protein can be obtained from a few different processes that generate end products consisting of varying levels of the constituent seed proteins. The major storage proteins in *Brassica napus* (canola) seed are cruciferin and napin, which comprise 85%–90% of the total proteins. Also present in small amounts are structural proteins and metabolic proteins. Cruciferin, an 11S globulin heteromer of 300–350 kDa, is the predominant seed storage protein. Napin, a 2S albumin of 14–16 kDa, is present in lesser amounts compared to cruciferin [5,6]. Many of the protein products obtained from canola/rapeseed using existing technologies are mixtures of these two (or more) protein types in different ratios. Since cruciferin and napin differ in many ways including amino acid composition, molecular structure, size and physico-chemical properties, the functional properties they exhibit under given conditions are different. Therefore the properties and functionalities of canola protein products may differ depending on the levels of cruciferin and napin in the product.

Solubility is a key functional requirement of food-based proteins. The processing history of protein products has a strong influence on the solubility characteristics of the final product. Alkali extracted and acid precipitated canola proteins showed depressed solubility around the pH that was employed in protein recovery, e.g., pH 3, 4 or 5 depending on the process. Solubility can be improved by hydrolysing the proteins. Napin-rich protein products show solubility values >90% in the range of pH 2 to 10, which is a unique characteristic for a plant (seed) protein. Cruciferin-rich protein products show depressed solubility compared to napin-rich protein products in this pH range [5].

The ability to emulsify oil (or other nonpolar molecules) without separating under a variety of storage and processing conditions is another key property of food proteins. Applications for proteins with good emulsifying properties exist in both food (liquid foods, emulsion-type processed meats, sauces) and non-food (e.g., personal care products) products. Canola protein isolates that contain both cruciferin and napin generally have limited emulsifying ability comparable to soy protein isolates. Low level of peptide bond disruption (3.1% to 7.7% degree of hydrolysis) improves oil emulsifying capacity (EC) and emulsion stability (ES). Hydrocolloids combined with high pH values also improved the emulsifying abilities. Canola napin exhibits poorer emulsifying properties than 11S proteins [5,6,7]. This means napin may negatively contribute to the emulsifying properties in canola protein products containing both cruciferin and napin. Canola protein isolates which are rich in cruciferin exhibit higher emulsion capacity than isolates which are rich in napin [8].

Heat-induced gel formation is a requirement of food proteins to create structure in thermally processed foods. This is not a strong functional aspect of canola protein isolates as the phenolic compounds present interfere with gel network formation [5]. Napin-rich products generate weak gels with intensive syneresis compared to cruciferin products, and this may be correlated with the high thermal stability of the napin molecular structure that has four disulfide bonds [5]. Cruciferin has a stronger tendency to form heat-induced gels than napin. Gel quality and properties generated from cruciferin-rich protein products can be improved by combining with hydrocolloids [5,6].

The ability of proteins to stabilize air–water interfaces to create foams is another requirement of food proteins. Napin-rich protein products (0.5% to 5% *w/v*) have exceptionally high foam forming ability and also stabilizing ability in a wide pH range (pH 3 to 10) in comparison to cruciferin-rich products [5,6].

Both Supertein™ and Puratein^®^ (BurconNutraScience) have generally recognized as safe (GRAS) status from the US Food and Drug Administration (FDA) for food use [9]. The proposed food categories that cruciferin-rich and napin-rich proteins products can be used include a wide range of bakery products, fruit and vegetable juices and flavoured drinks, egg substitutes, and processed meat products. In addition, Isolexx™ which is a canola protein product of *TeuTexx Proteins* has European Food Safety Authority (EFSA) approval under the novel food category [10].

### 2.3. Nutrition

#### 2.3.1. Protein Quality

The nutritional profile of canola protein would play a pivotal role in determining its suitability as a food ingredient. Canola protein is well balanced in essential amino acids and rich in sulfur-containing amino acids, which is mainly because of the comparatively high level of cysteine of napin. As canola proteins are almost exclusively used for animal feed, knowledge of their nutritional value to humans is quite limited. In a randomized cross-over intervention study in humans of a canola protein isolate containing cruciferin, napin and lipid transfer proteins, the Protein Digestibility Corrected Amino Acid Score (PDCAAS) was found to be 0.86, similar to the soy protein isolate [11]. True ileal digestibility of this product is reported as 84% while egg and milk protein reported 94% and 95%, respectively.

Combining digestibility data and AA profile, the calculated protein digestibility corrected amino acid score (PDCASS according to FAO/WHO 1989 standards [12]) of napin-rich Supertein™ and cruciferin-rich Puratein^®^ (BurconNutrascience) were 0.61 (61%) and 0.64 (64%), respectively [9]. Improved values of 0.83 and 0.71, for Supertein™ and Puratein^®^, respectively can be obtained when calculated according to updated FAO/WHO/UNU guidelines in 2002 [13] (considering reference amounts of specific amino acids and the requirements by age groups of children 1–2 years and 3–10 years). The limiting amino acid of these protein products are phenylalanine and tyrosine for Supertein™ and lysine for Puratein^®^ [9].

Evaluation of the above mentioned napin- and cruciferin–rich protein products in a 13-week rat feeding study, suggest usage level of the proteins with no-observed-adverse-effect-level (NOAEL); 11.24 g/kg BW/day for males and 14.11 g/kg BW/day for females for cruciferin-rich Puratein^®^ and 12.46 g/kg BW/day for males and 14.95 g/kg BW/day for females for napin-rich Supertein™ [9].

Safety assessment of the canola product Isolexx™ by EFSA reported that PDCAAS value for this product is similar to soy protein products, has low concentrations of antinutrional factors, and an absence of toxicologically relevant effects in sub-chronic studies with rats, making the protein a suitable candidate for the novel food ingredient category. It was estimated that “heavy” adult consumer intake (mean +2SD) of Isolexx™ would be 2.2 g/kg BW per day, 4–6 year old group mean intake of 3 g/kg BW per day and the 95th percentile intake of 4.73 g/kg BW per day [10].

#### 2.3.2. Allergenicity

The 2S seed storage proteins of Brassicaceae family plants are reported to consist of molecules that can trigger an immunogenic response by sensitive individuals. Both napin (most potent) and cruciferin (less potent) have been identified as allergenic proteins of yellow mustard (*Sinapis alba*) a close relative of canola. The napin isoform Bra n 1 (Napin BnIII, napin nIII or napin 3; P80208, 2SS3_BRANA) of *B. napus* is reported to cross-react with children that are sensitive to mustard and also have some form of food allergies [14]. Currently, no information is available on the effect of processing on the allergenic potential of canola napin. Considering the recognition of mustard as an allergen in EU countries and Canada, it has been recommended that canola protein-containing foods need to be appropriately labelled to indicate for potential allergenicity [9,10]. Allergenic potential of canola protein is a factor that cannot be excluded in the products derived from them.

#### 2.3.3. Bioactive Peptides

Peptide mixtures and hydrolysates derived from canola protein have been reported to possess a range of biological activities that could have beneficial health effects in humans. One study showed strong evidence for the ability of canola peptides to inhibit angiotensin I-converting enzyme that can interfere with Renin-angiotensin cascade, thereby lowering the blood pressure of hypertensive mice [15]. In addition, antioxidant, antidiabetic, anorexigenic, anticancer, antiviral, hypercholesterolemic and bile acid binding activities have been reported for peptides and hydrolysate fractions generated from canola proteins [5,7].

### 2.4. Breeding

There is the potential to use genomics and breeding techniques to improve canola meal for protein extraction and to increase bioavailability. However, it is not an option to improve protein at the expense of yield or oil content, and a very clear value to the market would need to be determined.

At first glance, it would appear that the current amino acid profile is relatively balanced. However, there is consensus that natural variability exists in *Brassica* that can be exploited to alter or improve amino acid composition for end-use markets, and genome editing techniques also have applicability. Increasing canola protein bioavailability for children as well as enhancing bypass amino acid composition/protein for ruminants are opportunities. As stated above, a robust evaluation is essential to understand the targets and economic value of these and other opportunities. As such, it would be prudent to target improvements in protein content first, and then look at protein types (cruciferin and napin) and their ratio. Reductions in fibre and glucosinolates could be a third goal. A goal of 1% increase in protein content each year may be realistic and achievable.

## 3. Discussion

### 3.1. The Opportunity

- Challenges aside, as food companies search for alternatives to soy protein and new plant proteins, an opportunity for canola emerges in the escalating demand for alternative protein sources.
- It is known that canola protein has a well-balanced amino acid profile, with a PDCAAS (canola protein products close to commercial entry level) that is very competitive with other plant proteins currently on the market. However, given the movement of the FAO to the DIAAS (Digestible Indispensible Amino Acid Score) method of evaluation of protein quality, it would be useful to have this measurement for canola protein.
- Canola has brand recognition as a healthy oil that is widely used and accepted in food processing and in the home. This brand acceptance should extend to the protein, although there may be GMO concerns with the protein that we do not see with the oil.
- Positioning of canola protein is very important, whether it be as a concentrate or isolate, and used in food or aquaculture. Unquestionably, the price of the canola protein product will be an important factor in its positioning.
- Opportunities also lie in the potential co-products from canola protein fractionation. These include: hull fibre, higher value protein fractions, lignin, phytic acid, polyphenols and canolol. An accurate techno-economic model is needed to evaluate all the opportunities for canola proteins and co-products.

### 3.2. The Challenge

- The biggest factor determining the success of canola protein as a food ingredient is being able to bring a product to market at a competitive price. The first step in this process is a thorough understanding of the potential market, including: market size, value of the product, price of feed stock, *etc*. Canola proteins will need to move into higher value markets (or the high volume markets, e.g., breakfast cereals) so the price and market size for higher value proteins need to be accurately assessed. In addition, price targets for production need to be identified.
- It would be necessary to identify the competitive advantage of canola proteins and develop strategies to market these competitive advantages effectively.
- Availability of a suitable feed-stock for protein extraction is a challenge. Conventionally processed canola meal which has gone through a desolventizer-toaster is not an efficient starting material for protein recovery. Currently, there isn’t a viable oil extraction technique that can replace this process without using solvent. Possible solutions are a low temperature desolventizer or vacuum desolventizer, but these would require expensive retrofitting of canola processing plants. Expeller pressing is an alternative oil extraction process, but if a cold-pressing process is used, the meal that is generated is too high in residual oil. Expeller pressed meal with low oil content has highly interacted protein somewhat similar to desolventizer-toasted meal. Although cold-pressed meal can be utilized for further recovery of oil, protein and other co-products, the economics of the technology and products needs to be competitive.
- Current technologies for obtaining canola products target generating protein ingredients to replace widely used proteins, especially those of animal origin. This approach limits options of using existing feed stock materials because some of the delicate functional properties of the final product become a key consideration. With the changing landscape of protein-rich products and how they are consumed (e.g., emulsion-type meat products with plant proteins *vs*. protein enriched, non transparent drinkable products), technologies need to be developed to use existing canola protein feed stock.

## 4. Workshop Topics and Presenters

- Opportunities and Challenges for Bringing Protein to Market.
  Martin Schweizer, BurconNutrascience MB Corp., Winnipeg, MB, Canada
- Processing of Canola Protein—Current Challenges and New Technologies.
  Rick Green, POS BioSciences, Saskatoon, SK, Canada
- Canola Protein—Functionality and Nutrition.
  Janitha P.D. Wanasundara, Agriculture and Agri-Food Canada, Saskatoon, SK, Canada
- Genomics and Breeding for Protein Improvement.
  Rob Duncan, University of Manitoba, Winnipeg MB Canada.
